# Acidic Gases Separation from Gas Mixtures on the Supported Ionic Liquid Membranes Providing the Facilitated and Solution-Diffusion Transport Mechanisms

**DOI:** 10.3390/membranes9010009

**Published:** 2019-01-05

**Authors:** Alsu I. Akhmetshina, Nail R. Yanbikov, Artem A. Atlaskin, Maxim M. Trubyanov, Amal Mechergui, Ksenia V. Otvagina, Evgeny N. Razov, Alla E. Mochalova, Ilya V. Vorotyntsev

**Affiliations:** 1 Laboratory of Membrane and Catalytic Processes, Nizhny Novgorod State Technical University n.a. R.E. Alekseev, 24 Minina str., Nizhny Novgorod 603950, Russia; aai-89@mail.ru (A.I.A.); nailyanbikov@gmail.com (N.R.Y.); atlaskin@gmail.com (A.A.A.); m.trubyanov@yandex.ru (M.M.T.); amalfatroucha@gmail.com (A.M.); k.v.otvagina@gmail.com (K.V.O.); mochalova_ae@mail.ru (A.E.M.); 2 Kazan National Research Technological University, 68 Karl Marks str, Kazan 420015, Russia; 3 Institute for Problems in Mechanical Engineering, Russian Academy of Sciences, 85 Belinskogo str., Nizhny Novgorod 603024, Russia; razov_e@mail.ru; 4 Department of Chemistry, N.I. Lobachevsky State University of Nizhny Novgorod, 23 Gagarin Avenue, Nizhny Novgorod 603950, Russian

**Keywords:** gas mixtures, supported ionic liquid membrane, hydrogen sulfide, carbon dioxide, natural gas treating

## Abstract

Nowadays, the imidazolium-based ionic liquids containing acetate counter-ions are attracting much attention as both highly selective absorbents of the acidic gases and CO_2_ carriers in the supported ionic liquid membranes. In this regard, the investigation of the gas transport properties of such membranes may be appropriate for better understanding of various factors affecting the separation performance and the selection of the optimal operating conditions. In this work, we have tested CH_4_, CO_2_ and H_2_S permeability across the supported ionic liquid membranes impregnated by 1-butyl-3-methylimidazolium acetate (bmim[OAc]) with the following determination of the ideal selectivity in order to compare the facilitated transport membrane performance with the supported ionic liquid membrane (SILM) that provides solution-diffusion mechanism, namely, containing 1-butyl-3-methylimidazolium tetrafluoroborate (bmim[BF_4_]). Both SILMs have showed modest individual gases permeability and ideal selectivity of CO_2_/CH_4_ and H_2_S/CH_4_ separation that achieves values up to 15 and 32, respectively. The effect of the feed gas mixture composition on the permeability of acidic gases and permeselectivity of the gas pair was investigated. It turned out that the permeation behavior for the bmim[OAc]-based SILM toward the binary CO_2_/CH_4_, H_2_S/CH_4_ and ternary CO_2_/H_2_S/CH_4_ mixtures was featured with high acidic gases selectivity due to the relatively low methane penetration through the liquid phase saturated by acidic gases.

## 1. Introduction

There is a high demand for the reduction of acidic gases across the world due to the strong need to deal with the global warming and the rapidly-increasing energy consumption. As long as petroleum, coal and natural gas are used as the primary global fuel, the production of CO_2_ is inevitable. Acidic gases such as CO_2_ and H_2_S contribute to pipeline corrosion and decrease the calorific value of the natural gas [1]; therefore their separation is of critical economic importance. However, the feed gas conditions of CO_2_ and H_2_S separation from various sources are different. For example, post-combustion flue gas has low CO_2_ concentrations and low feed pressures, while natural gas or syngas have much comparable CO_2_ concentrations and feed pressures. In this regard, membrane separation is considered as an emerging technology and has numerous advantages over traditional CO_2_ and H_2_S adsorption and absorption methods, such as the low energy consumption, the operational simplicity, and the low environmental impact [2], which make it particularly attractive in small- and medium-scale applications [3]. The development of high-performance СН_4_/СО_2_ separation membranes with both high permeability and selectivity has been a current issue in recent years [4], while the use of ionic liquids (ILs) in membrane separation processes is one of the fastest growing research topics [5,6,7,8,9]. Many types of membranes and membrane processes containing ILs have been reported, including supported ionic liquid membranes (SILMs), polymerized ionic liquid membranes, polymer/IL gel membranes. Among these diverse gas mixtures, the most known (described) one seems to be the CO_2_/CH_4_ separations, associated respectively with the purification of natural gas [10].

Previous studies using SILMs have shown them as promising alternatives for the separation of CO_2_/CH_4_ gas pair [11,12]. Mixed-gas permeabilities and selectivities for the CO_2_/CH_4_ have been determined by Scovazzo et al. [13], and it has been observed that the selectivity for a gas mixture slightly decreases when compared to the ideal selectivity. The investigations have shown that room temperature ionic liquid (RTIL)-membranes based on emim[BF_4_], emim[dca], and emim[CF_3_SO_3_] can operate at CO_2_-partial pressures of at least 207 kPa without any decrease in their separation ability (>106 days). Neves et al. [14] have studied the effect of water vapour in different gas streams of CH_4_ and CO_2_ in SILMs with RTILs based on the 1-alkyl-3-methylimidazolium cation. The presence of water vapor in a gas stream increased the gas permeability but decreased their CO_2_/CH_4_ selectivity significantly. Moreover, the hydrophobic polyvinylidene fluoride (PVDF)-based membranes were more stable than those based on the hydrophilic support. SILMs turned out to be especially selective for CO_2_/CH_4_ separations, and the results have been above the Robeson upper bound correlation. In study [15] SILMs have been prepared by impregnating the pores of *γ*-alumina inorganic supports with pure RTILs along with RTIL and organic salt with amine group functionality mixtures. Nevertheless, they were found to be unsuitable for gas separation at high pressures and their separation performance suffered at high temperatures due to decrease in gas solubility. SILM consisting of α-alumina support impregnated with the IL emim[FAP] [16] have shown high CO_2_ absorptive capacity and selectivity (9.69); but the CO_2_/CH_4_ mixed gas permselectivity α was found to be much lower (1.15) than the ideal permselectivity (3.12) because of the higher CH_4_ diffusivity compared to CO_2_. The performance of the SILM has been negatively affected by the presence of water. The dense polymer SILMs [17] on polysulfone support prepared for the selective separation of CO_2_ at high pressures gave promising results for CO_2_/CH_4_ separation. Membranes based on C_4_mim[NTf_2_] and DIP-C_4_mim[NTf_2_] ILs have shown the highest CO_2_/CH_4_ selectivities (70 and 63) and CO_2_ permeabilities (11.5 and 13.8), respectively, behaving similar or even better than the reported polysulfone blends. No IL loss have been observed for SILMs at 10 bar after 12 h, indicating that the synthesized dense polymer SILMs are stable at high pressures for long durations [17]. 

**Figure 1:**
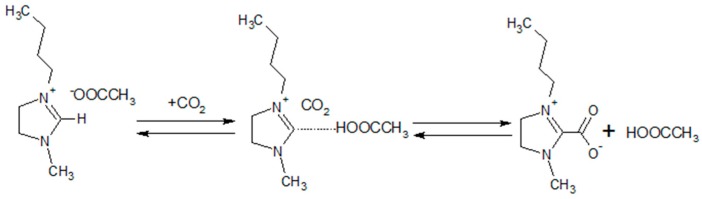
Possible reaction of bmim[OAc] and CO_2_ [35,36].

Apart from RTILs, task-specific ILs are frequently used as CO_2_ carriers to facilitate the CO_2_ transport along with the solution-diffusion mechanism. Fixed-site-carrier facilitating the transport of CO_2_ has been reported for many different membrane compositions [18,19,20,21,22,23,24,25] and it generally requires humidified conditions. Amine-containing polymers and small molecules are the most common CO_2_ carriers [26]. Previously, Quinn et al. have prepared facilitated transport membranes (FTMs) by immobilizing salt hydrates in a microporous Celgard membrane [27]. At 323 K and CO_2_ partial pressure of 28 torr, CO_2_ permeability and CO_2_/CH_4_ selectivity were 1720 Barrer and 120, respectively. However, as the CO_2_ partial pressure increased, both CO_2_ permeability and selectivity dropped rapidly, resulting in CO_2_ permeability of 176 Barrer and selectivity of 14 at the CO_2_ partial pressure of 1 atm. The CO_2_/CH_4_ mixture studied by Hanioka et al. [28], where the highly stable (during more than 260 days) and selective membrane for CO_2_ separation has been obtained using SILM facilitated by the amine-terminated IL immobilized into the hydrophilic polytetrafluoroethylene (PTFE). At 2.5 kPa of CO_2_, bis(trifluoromethylsulfonyl)imide and trifluoromethanesulfone-based SILMs achieved the CO_2_/CH_4_ selectivity of approximately 100 and 120, respectively. Recently CO_2_ reactive amino acid ionic liquid [29,30,31,32,33] based FTM has been developed with exceptionally high levels of CO_2_ permeability regardless of humidity which is a particularly novel feature. Besides amine-tethered ILs, another class of molten salts able to form N-heterocyclic carbenes [34] has been attracting wide attention due to the permanence of physical-chemical properties during the experiments, low viscosity, stability and high absorption capacity. Rogers [35] and Maginn [26] have reported that the basic anions such as acetate may remove the acidic proton on the C(2) position of cation with subsequent formation of N-heterocyclic carbine, which reversibly react with acidic gases (Figure 1). 

In this context, Santos et al. have obtained the results of CO_2_ separation on the acetate-based SILMs that was near the Robeson upper bound corresponding to the best polymeric materials [34]. The promising acidic gases separation performance was achieved on the Al_2_O_3_/TiO_2_ tubes with immobilized emim[OAc] [37]. To the best of our knowledge, no study has investigated the СО_2_/Н_2_S or СН_4_/СО_2_/Н_2_S mixed gas separations using the facilitated IL-based membranes. Therefore, there is high demand on future studies of the performance and stability of the membranes under СН_4_/СО_2_/Н_2_S mixed gas conditions which will be beneficial for the deployment of the membranes in real applications.

Due to the limited number of publications on the facilitated separation of acidic gases from gas mixtures using the SILMs, it is reasonable to investigate the gas transport behavior of task-specific carboxylate ionic liquids, such as 1-butyl-3-methylimidazolium acetate, toward CO_2_/CH_4_, H_2_S/CH_4_ and СН_4_/СО_2_/Н_2_S mixtures. In this work, the permeability of pure CO_2_, H_2_S and CH_4_ through the SILMs consisting of fluorinated polymer with immobilized 1-butyl-3-methylimidazolium tetrafluoroborate and 1-butyl-3-methylimidazolium acetate were measured in membrane gas separation setup with continuous sweeping gas supply. These measurements were performed in order to evaluate the distinctions in gas transport behavior of the ILs dissolving the acidic gases via the physical and chemical mechanisms. Permeabilities and selectivities of membranes for CO_2_/CH_4_, H_2_S/CH_4_, and CH_4_/CO_2_/H_2_S gas mixtures were compared with the respective single gas permeabilities and ideal selectivities; the stability of the SILMs under operational conditions was evaluated gravimetrically and via the contact angle data.

## 2. Materials and Methods

In this study, commercially available flat sheet porous microfiltration tetrafluoroethylene-vinylidene fluoride composite membrane named as MFFK-1 with pores size 150 nm and thickness 100 μm, purchased from Vladipor JSC (Vladimir, Russia) was used as a polymeric support. The membrane was composed of tetrafluoroethylene-vinylidene fluoride copolymer of F42L (upper layer) and the nonwoven polypropylene bottom layer. To prepare the binary and ternary gas systems the high purity (99.9 vol.%) methane, carbon dioxide (99.99 vol.%) and hydrogen sulfide (99.9 vol.%) purchased from Monitoring (Russia) were used. Ionic liquids bmim[BF_4_] and bmim[OAc] were purchased from Sigma Aldrich Group (St. Louis, MO, USA). Their physical properties are given in Table 1. All ILs were dried under vacuum for 24 h, at a point the water content found by Karl-Fischer titration (Coulometer 831 KF, Metrohm, Switzerland) less than 0.2% (*w*/*w*).

For the preparation of supported ionic liquid membranes, a vacuum method was used. The polymeric support was fixed into a desiccator and was evacuated at 10^−5^ mbar for at least for 2 h in order to remove air from pores. The ionic liquid was then introduced into a desiccator using a syringe, while maintaining the vacuum inside the desiccator, and spread on the surface of the membrane (for 2 h). Once samples were then taken out, the excess IL was removed by wiping with absorbing tissue. To determine the amount of IL immobilized (uptake), the membrane was weighed before and after immobilization procedure. The thickness was also determined before and after immobilization.

The membranes surface wettability was evaluated by contact angle measurements. The static contact angle was measured using an optical contact angle measurement system. A droplet of testing liquid was placed on the membrane surface and an image of drop shape was obtained. The optical system apparatus consisted of a light source, an adjustable stage, and a USB optical microscope. The microscope (Chuo Seiki, TS-H, Kumamoto, Japan) was fixed on an adjustable microscope mount. A digital image of the drop shape was made using a CCD camera interfaced to the microscope. Image J^®^ software with Dropsnake plugin was used for the calculation of the contact angle value. The contact angle value for each testing liquid was calculated as an average of 5 measurements of different positions for each sample. An electronic balance (Shimadzu AUW-220D, Kyoto, Japan) with the standard uncertainty of 0.01 mg was used for the gravimetric measurements.

The feed gas mixtures CH_4_/CO_2_, CH_4_/H_2_S and CH_4_/CO_2_/H_2_S were prepared in sealable gas container by static volumetric method. The CO_2_ and H_2_S content in binary system were 15 ± 0.05 vol.% and 5 ± 0.05 vol.% (mixed with 85 ± 0.05 or 95 ± 0.05 of CH_4_), respectively. The CO_2_ and H_2_S content in ternary system were 18.2 ± 0.05 vol.% and 5.3 ± 0.05 vol.%, respectively. Gas mixture components were filled in the preliminary vacuumed container to reach the appropriate ratios. The gas mixtures composition verification was performed by gas chromatography (GC) method. The gases were introduced into the flow-through sampling valve of the gas chromatograph (Chromos GC-1000, Chromos Ltd., Dzerzhinsk, Nizhny Novgorod region, Russia) for the GC analysis. During the analysis the components of the permeate sample are separated in the chromatographic column (Porapak Q, 60/80 mesh, 353 K, 2 m × 3 mm i.d. stainless steel tube) in isothermal conditions and detected by the thermal conductivity detector (TCD, 100 mA, 100 °С). Carrier gas flow controller serves to supply the required flows of the carrier gas (Helium 99.9999+%). The low limit of detection was 4 ppmv for methane, hydrogen sulfide and carbon dioxide. The total analysis time was not more than 300 s.

**Figure 2:**
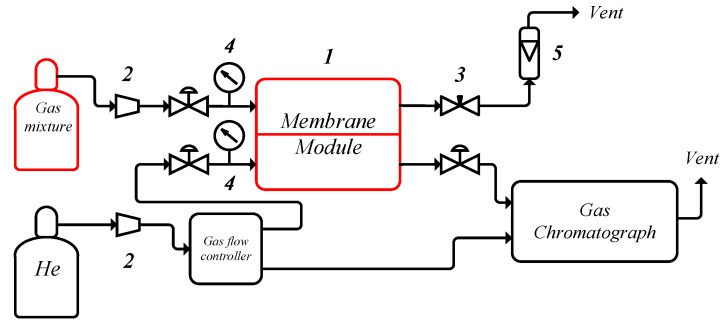
The principal scheme of the experimental setup.

The principal scheme of experimental setup is shown in Figure 2. The SILM with active area 4.9 cm^2^ was placed on a PTFE disk support and sealed in a stainless-steel membrane module 1 using the Viton O-ring. The gas mixture is continuously supplied from the cylinder to the feed side of a membrane module (1) through a pressure regulator (2) with a constant pressure maintained at (200 ± 5) kPa. Pressure in the system was monitored by manometers (4). The gas permeated through the membrane is removed by a helium flow, which is also used as a carrier gas for GC system. Retentate flow rate is controlled by a needle valve (3) and monitored with a flow meter (5). Permeate content determination was performed by gas chromatography method. «Ex-situ» qualitative analysis of the permeate sample was carried out on the gas chromatograph-mass spectrometer (GCMS) QP2010Plus (Shimadzu, Kyoto, Japan) with a vacuum sample inlet system through automatic injection valve (Valco Instruments Co Inc., Houston, TX, USA). During the analysis the components of the permeate sample are separated on an Agilent capillary column Select for Permanent Gases/CO_2_ with set of two parallel columns: CP-Molsieve 5 Å for permanent gases and PoraBOND Q for CO_2_ analysis in accordance with the following temperature program: holding at 323 K (10 min), 323–423 K (20 K/min), holding at 423 K (5 min), carrier gas-helium (99.99999 vol.%). Quantitative analysis was performed by the method of absolute calibration. Reaction products were identified with the help of NIST-11 database of mass spectra and GCMS Real Time Analysis^®^ software.

The stability of SILMs was determined by the gravimetric method. The weights of SILMs before and after N_2_ transport performances were compared. For the pressure values tested (1 bar), the distinctions in membranes weight are within the range of instrumental error.

## 3. Results and Discussion

### 3.1. Membranes Characterization

The hydrophobic tetrafluoroethylene-vinylidene fluoride copolymers (MFFK-1) with pore size equal to 150 nm was investigated as a supporting membrane material. This type of supporting material has been selected bearing in mind the results of the SILMs gas transport properties reported in our previous work [40]. According to those results, the highest values of acidic gases separation have been observed for the combination of MFFK-1 supporting material and bmim[BF_4_] liquid phase. It is noteworthy that the permeability of gases through the IL phase significantly exceeded that of through the dense polymeric support.

Compatibility of a supporting material with a liquid phase is a key factor determining the stability and operating properties of the SILMs. The interactions that may occur between the matrix and the IL contribute to the SILM stability, which can be estimated using contact angle measurements. The wettability investigations of hydrophobic MFFK-1 membrane by bmim[BF_4_] and bmim[OAc] have been carried out using the sessile drop method. The contact angles for the ILs are listed in Table 2 together with the calculated capillary pressures. According to this data, the fluorinated IL bmim[BF_4_] had remarkable affinity to the MFFK-1 surface composed of the fluorinated polymer. The contact angle of bmim[OAc] on MFFK-1 was slightly higher pointing to poorer interactions between the IL and the membrane. In general, it was found that the membrane has displayed a significant affinity toward both ILs.

**Figure 3:**
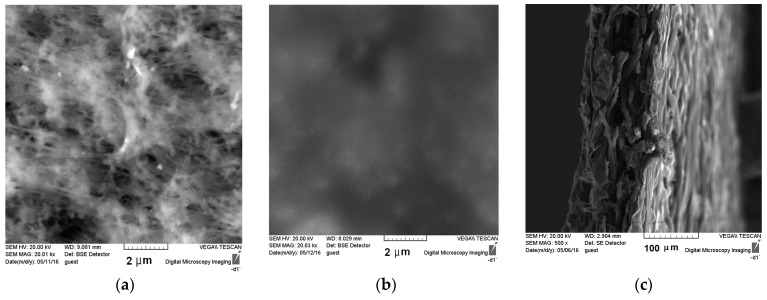
Scanning electron micrographs of the polymeric membrane surface of MFFK-1 before (**a**) and after (**b**) immobilization of bmim[OAc]; (**c**) the cross-section of the membrane.

In addition, the distribution of bmim[OAc] on the MFFK-1 surface was observed using scanning electron microscope (SEM) technique. Filling of the porous support by bmim[BF_4_] was examined in previous work [40]. As it can be seen from Figure 3, SEM micrographs of the membrane surfaces together with gravimetric measurements, contact angle data, and stabile gas transport properties revealed the compatibility of the supporting material and complete filling of the pores.

The stability of the supported ionic liquid membranes depends on the capillary holding force, the supporting material pore sizes, and the viscosity of a liquid phase. This requires that during gas separation processes, the total amount of IL immobilized should remain constant inside the pores of the support. The minimal pressure required for desorption of the impregnated phase from support pores is calculated by the Young-Laplace equation at the steady state condition:

*p_c_* = (2*σ*·cos*θ*)/*r* (1)

where *p_c_* is the capillary pressure in Pascals, *σ* is the surface tension in N/m, *θ* is the contact angle in degree, and *r* is the average pore radius in meters [42].

**Figure 4:**
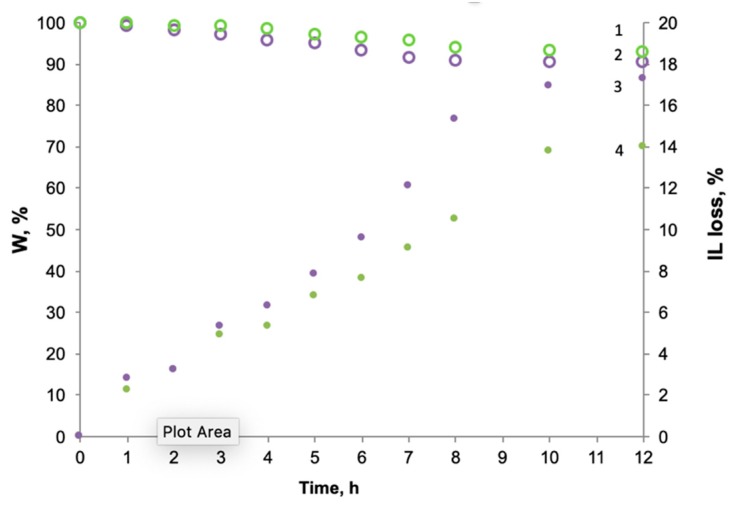
Membrane weight fraction *W* (1—bmim[OAc], 2—bmim[BF_4_]) and an ILs loss (3—bmim[BF_4_], 4—bmim[ace]) and as a function of time.

Stability measurements have been carried out gravimetrically for 12 h at the N_2_ transmembrane pressure equal to 1 bar. The membrane weight together with the liquid phase loss as a function of time is depicted in Figure 4. It is observed that the membrane weight loss is only owing to the partial desorption of an IL immobilized within the supporting material. Both ILs have exhibited the similar stability behavior, so the gradual decrease of liquid phase has been noticed during the 10 h followed by a further stabilization. In case of bmim[OAc] the membrane weight diminished to approximately 93% accompanied by the IL loss equal to 14%. The SILM impregnated by bmim[BF_4_] loses 17.3% of the IL, while the membrane weight change was about 10%.

The obtained results have been compared with the data reported in literature [14,17,43]. Neves et al. compared the influence of the support hydrophobicity on the stability of the SILMs composed of fluorinated ILs [14]. The weight loss of hydrophilic membranes was more pronounced reaching 11–13%, while the hydrophobic ones were characterized by 1.5–7% decrease of the SILM weight. Alkhouzaam et al. evaluated the stability of polysulfone-supported ionic liquid membranes applying a pressure difference of 10 bars, for which the IL weight loss did not exceed 30% [17]. Zhao et al. studied the effect of the pore size and the transmembrane pressures on the polysulfone SILMs stability and reported the 20% loss of IL at 1 bar pressure difference [43]. In general, the results on the SILMs stability studied in this work have correlated with literature, taking into account the distinctions in the membranes composition, pore sizes and transmembrane pressures.

### 3.2. Single Gas Permeability

**Figure 5:**
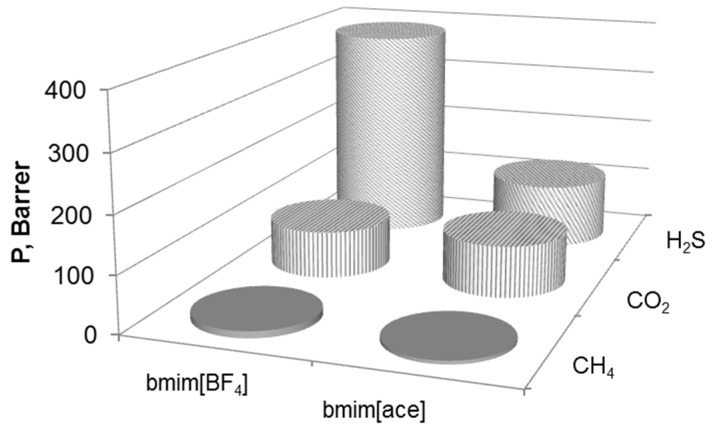
CH_4_, CO_2_ and H_2_S gas permeability (Barrer) of SILMs based on bmim[BF_4_] and bmim[OAc].

The results of the single gas (CH_4_, CO_2_ and H_2_S) permeabilities through the prepared SILMs are shown in Figure 5 for both bmim[BF_4_] and bmim[OAc] ionic liquids. It can be observed that pure H_2_S shows the highest permeability (~380 Barrer) through the MFFK-1 doped by bmim[BF_4_] than pure CO_2_ and CH_4_ (~80 and 12 Barrer, respectively). In terms of MFFK-1 containing bmim[OAc] the permeabilities are much lower, taking on the values of ~110, 90 and 6 Barrer, respectively.

Explanation of different gas transport behavior in the bmim[BF_4_] and bmim[OAc] is based on two different mechanisms of acidic gases penetration across the SILMs. In case of bmim[BF_4_] the transport of CO_2_ and H_2_S undergoes in accordance with the physical absorption of gases in the liquid phase followed by the diffusion on the other side of the membrane. The permeation of the non-polar methane molecules through both ILs also takes place within the solution-diffusion mechanism. Therefore, the gas transport behavior of the SILMs containing bmim[BF_4_] and the methane transport through both ILs may be analyzed via the solution-diffusion mechanism, according to which the permeabilities of the individual gases are considered in terms of solution-diffusion model defined as a product of the solubility coefficient and the diffusivity coefficient:

*P* = *D* × *S* (2)

where *P* is permeability in mol·m/(m^2^·s·Pa), *S* is the solubility coefficient in mol/(m^3^·Pa) and *D* is the diffusivity coefficient in m^2^/s. The diffusivity coefficients for the SILMs were calculated by an equation proposed by Morgan et al. [44] for imidazolium-based ILs:

(3) $$D=3.7\times 10^{-4.77}\frac{1}{\mu_{2}^{0.59}V_{1}\rho_{2}^{2}},$$

where *D* is the diffusivity of gases in ILs (m^2^·s^−1^); *µ*_2_ is the IL viscosity (Pa·s) at 298 K (Table 1); *V*_1_ is the gas molar volume (m^3^·mol^−1^); and $\rho_{2}^{2}$ is the density of the IL (kg·m^−3^). As shown in Table 3, among the gases the lowest diffusivity coefficient was in case of CO_2_ penetration caused by the largest dimensions of the molecule (the Lennard-Jones diameter) whereas the CH_4_ and H_2_S diffusion was almost identical. Owing to the higher viscosity of bmim[OAc], the calculated diffusivity coefficient for this IL was approximately 3-fold lower than this for bmim[BF_4_].

The solubilities of the different gases (*S_calc_*) in the IL at the feed conditions (298 K, 0.2 MPa) were obtained using Equation (2), in which the permeabilities were preliminarily divided by the porosity of the membrane (80%), due to gas permeation occuring only through the pores filled with IL. Alternatively, known from the literature data [16] the solubilities of gases in the ILs (*S_lit_*) given in mole fraction *x* were converted to the required units and compared with the calculated data:

(4) $$S_{\mathrm{lit}}=\frac{x}{p\left(1-x\right)\frac{M_{\mathrm{IL}}}{\rho_{\mathrm{IL}}}},$$

where *M_IL_* is the molecular weight of the IL, *ρ_IL_* is the density of the IL and *p* is the feed pressure.

The permeation test results for bmim[BF_4_] were almost identical to those described in our study [40], but the remarkable differences were found compared with the solubility data known from literature. In particular, whereas the methane solubility coefficients were similar to the *S_lit_*, the CO_2_ and H_2_S solubilities were in 4.25 and 4.45 times lower. Explanation of the dramatic diminishing of ILs sorption properties toward the acidic gases may be the consequence of water presence in bmim[BF_4_], which is highly hydroscopic. Indeed, during the preparation of the SILM, it was exposed to air resulting in the presence of 0.168% (*w*/*w*) of water. This may lead to the reduction in gas solubility and ultimately to the reduction in the permeance. In the acetate-based IL the calculated solubility of methane differed from *S_lit_* insignificantly. On the other hand, the acetate-based IL interacts chemically with acidic gases (CO_2_ and H_2_S) yielding the formation of the adducts [47]. Moreover, the facilitated transport mechanism of acidic gases across bmim[OAc] favors low concentrations and decreases drastically with increase of transmembrane pressure. In particular, Zhang et al. have reported the superior permeabilities of the acetate-based SILMs varying in a range about 1000–7000 Barrer at transmembrane pressures 0.1–0.5 bar. In the present work, the transmembrane pressure was equal to 1 bar, which, probably, dramatically diminished to the permeability of acidic gases. The distinctions in permeabilities of both acidic gases was less significant than in bmim[BF_4_] indicating the chemical binding of gases. This observation is additionally proved by the comparable solubility values of carbon dioxide (0.272 mole fraction [48]) and hydrogen sulfide (0.255 mole fraction [49]) in bmim[OAc].

The chemisorption of acidic gases in bmim[OAc] has resulted in an unsubstantial difference between the selectivity of CO_2_/CH_4_ and H_2_S/CH_4_. And vice versa, for the SILMs impregnated by bmim[BF_4_] the distinctions concerning those two acidic gases separation were remarkable. The ideal selectivity as the ratio of the permeances of CO_2_ and H_2_S pure gases over the CH_4_ are presented in the Table 4. The measured difference between the ideal selectivity values can be attributed to the solubility selectivity of bmim[BF_4_] IL, which is higher for hydrogen sulfide and is lower for CO_2_. The ideal selectivity for CO_2_/CH_4_ and H_2_S/CH_4_ will be compared next with the mixed gas selectivity.

### 3.3. Mixed Gas Permeability

The mixtures of CH_4_/CO_2_ = 85/15% (*v*/*v*) and CH_4_/H_2_S = 95/5% (*v*/*v*) were also investigated for measuring the permeabilities and calculating the separation factor, as shown in the Table 4. Compared to the single gas permeation results, the CO_2_ and CH_4_ permeabilities of the MFFK-1 bmim[BF_4_] membrane measured in the mixed gas test were much lower. The separation CH_4_/CO_2_ and CH_4_/H_2_S binary mixtures across the SILMs impregnated by bmim[BF_4_] was generally determined by the solubility selectivity and was comparable with the separation of ternary mixture (Table 4). This is reflected in the fact that the selectivities of CO_2_/CH_4_ and H_2_S /CH_4_ separation were equal to 8.7 and 23.4 in the binary mixtures and 8.1 and 15.9 in the ternary mixture.

Meanwhile in the case of bmim[OAc] the value of CO_2_ permeability doubled to 180 Barrer, which led to a sharp increase of CO_2_/CH_4_ selectivity (~96.9) in comparison with the ideal selectivity. As mentioned in [47], the facilitated transport membranes achieve the highest separation results at lowest concentrations of the active penetrants, therefore individual gases testing shows the modest results compared to the gas mixtures. Interestingly, the separation factors for both acidic gases in bmim[OAc] differ insignificantly as in case of individual gases. The results of the CH_4_/H_2_S mixed gas separation revealed the similar behavior as in the case of CH_4_/CO_2_. The effect of the incorporation of bmim[OAc] IL in the SILM is more pronounced, causing the double growth of the mixed H_2_S permeability up to 205 Barrer and consequently the sharp increase of the H_2_S/CH_4_ selectivity up to 102.9. Hence, it is clear that the incorporation of bmim[OAc] in SILM is very promising for the removal of the both acidic gases from the gas streams.

In the separation of the ternary CH_4_/CO_2_/H_2_S = 76.5/18.2/5.3% (*v*/*v*/*v*) gas mixture it was found that MFFK-1[bmim][BF_4_] membrane had better permeability to H_2_S (~140 Barrer) and CO_2_ (~69 Barrer) than to CH_4_ (8.6 Barrer). In case of MFFK-1 bmim[OAc] membrane the permeabilities to H_2_S and CO_2_ have close values (110 and 100, respectively), as for CH_4_ it converges to very low values.

The separation of the ternary mixture CH_4_/CO_2_/H_2_S has confirmed the previously noted patterns of the CO_2_ and H_2_S penetration. In bmim[BF_4_] the differences in CO_2_ and H_2_S solubilities resulted in the remarkable permeability variance, whereas the chemisorption in bmim[OAc] almost equalized the permeabilities of them. From this point of view, the gas transport behavior of both ILs for CH_4_/CO_2_/H_2_S mixture duplicates the results of binary mixtures separation and pure gases. At the same time, the particular phenomena was observed when separating the binary and ternary gas mixtures through the membrane immobilized with bmim[OAc], such SILMs were almost impermeable for the methane despite its high concentrations in gas mixtures. Most probably, the changes in the IL structure led to an extremely low solubility of methane in the CO_2_- or H_2_S-saturated ILs resulted in a negligible permeability values. This tendency was also noticed for the ternary mixture permeation. This point stipulated the high values of selectivity of the proposed membranes toward acidic gases.

### 3.4. Effect of Temperature on the H_2_S Transport

The effect of temperature on the CO_2_ penetration across SILMS has been widely addressed in the literature, incorporating reports on the membranes impregnated by either conventional ILs [5,6,7,10,12,13,14,15] or providing the facilitated transport ones [6,27,47]; therefore, the further studies of the SILMs were carried out for H_2_S-containing binary mixture in the temperature range 298–333 К. The membrane impregnated by bmim[BF_4_] was excluded from the study owing to a sufficient level of scrutiny of such SILMs.

**Figure 6:**
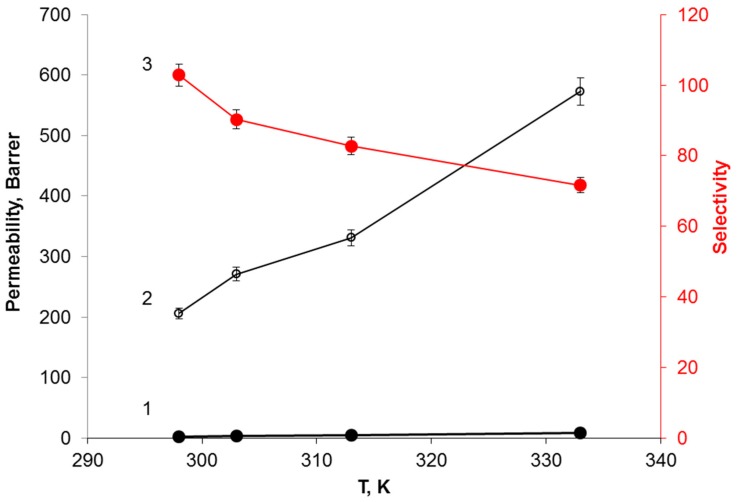
Effect of temperature on the permeability of CH_4_ (1) and H_2_S (2) across MFFK-1 [bmim][ace] membrane and on the selectivity of H_2_S/CH_4_ (3).

As illustrated in Figure 6, the permeability of CH_4_ and H_2_S increase with increasing temperature due to the acceleration of the diffusion rate. At the elevated temperatures, the viscosity of IL dramatically diminishes resulting in the enhanced gas diffusion. The permeabilities of CH_4_ and H_2_S increase from 2 to 8 Barrer and from 205 to 573 Barrer, respectively. It is noteworthy that, despite smaller dimensions of the H_2_S molecule, the permeability of H_2_S is doubled in the temperature range 298–333 К, whereas that of CH_4_ increases fourfold. This is most probably due to both decreasing of the physical solubility and weakening the chemical interaction between bmim[OAc] and H_2_S [47]. The selectivity of H_2_S/CH_4_ separation has undergone a significant decrease from 102.9 to 72. Thermodynamic factors together with weakening the complexation of H_2_S with the IL contribute to a notable decline in the separation efficiency of the membrane.

### 3.5. SILMs Performance Comparison

The gas separation performances of the SILMs containing bmim[BF_4_] or bmim[ace] reported in literature are represented in Table 4, including the results of this work. Those data correspond to both individual gases separation, and gas mixtures. It is worth drawing attention to the similarities between the SILMs containing bmim[BF_4_] studied in our work and the ones mentioned in [47]. Although the permeabilities of all gases in [47] were several times higher, the selectivities of CO_2_/CH_4_ and H_2_S/CH_4_ separation were consistent with our results. Other literature reference [50] reports the similar CO_2_/CH_4_ ideal selectivity, but notices the considerably higher results for H_2_S/CH_4_ separation. Owing to the approximately equal values of separation of gas mixtures and individual gases on the SILM impregnated by bmim[BF_4_], the relevant comparison of the pure gases is applicable to the gas mixtures as well. On the contrary, the permeabilities of the individual gases across bmim[OAc] as well as the selectivities were fairly low and were not comparable with the data given in work [49]. Only in case of hydrogen sulfide removal the selectivity values that were found for the binary and ternary mixtures were consistent with the literature data. The binary mixture separation has been slightly higher than that reported in the aforementioned work as well as for the ternary mixture. Most probably, diminishing the selectivity for ternary mixture is the result of the competitive absorption phenomena of the both acidic gases, which is not possible in binary mixtures. Removal of carbon dioxide from gas mixtures on bmim[OAc] was found to be more pronounced in our work than in literature. And obtained membranes might be used for the separation of above mentioned mixtures in cascades for better separation efficiency [50,51].

## 4. Conclusions

In this work, the membranes consisting of fluorinated porous polymer with immobilized 1-butyl-3-methylimidazolium tetrafluoroborate and 1-butyl-3-methylimidazolium acetate have been tested for pure CO_2_, H_2_S and CH_4_ as well for the binary mixtures of H_2_S/CH_4_, CO_2_/CH_4_ and the ternary CH_4_/CO_2_/H_2_S gas mixture.

The permeability of carbon dioxide (single) through the bmim[OAc]-based SILM has been approximately 2 times greater as compared to the SILM doped by bmim[BF_4_]. On the contrary, the ideal selectivity of H_2_S/CH_4_ has been much higher than that for facilitated transport membrane (containing bmim[OAc]). The opposite trend has been observed for the mixed gas transport across the facilitated transport membrane. The latter was found to exhibit a negligible permeability of methane in CO_2_- or H_2_S-containing mixtures resulting in remarkable high selectivity of CO_2_ or H_2_S separation. The gas transport behavior of bmim[OAc] for ternary mixture has duplicated the results of binary mixtures separation. In the case of bmim[OAc], the mass transfer of gases across SILMs may be enhanced by carrier-mediated reversible reactions.

From the results obtained in this work, it can be inferred that facilitated transport membranes containing the task-specific ionic liquids, which are able to form N-heterocyclic carbenes, is very promising media for the removal of the both acidic gases from the gas streams.

## Figures and Tables

**Table 1:** The physical properties and water content of bmim[BF_4_] and bmim[OAc].

Ionic Liquid	Density, kg/m^3^	Viscosity 10^3^, Pa·s	Water Content, ppm
bmim[BF_4_]	1200.7 ^a^	110.3	1679
bmim[OAc]	1055.0	343.3 ^b^	1713

^a^ [38]; ^b^ [39].

**Table 2:** Contact angle of ionic liquids on MFFK-1 and calculated capillary pressure.

IL	Contact Angle, °	Surface Tension, N/m	Capillary Pressure, bar
bmim[BF_4_]	44 ± 0.8	44.8 × 10^−3 a^	8.6
bmim[OAc]	49 ± 0.7	37.6 × 10^−3 b^	6.6

^a^ [41]; ^b^ [39].

**Table 3:** Diffusivities (*D*) of various gases in the ILs at 298 K, the gas solubility’s based on the literature (*S_lit_*) and calculated (*S_calc_*) data, and the gas permeabilities (*P*).

Gas	*P* 10^−15^ (mol·m/m^2^·s·Pa) ^a^	*D* 10^−11^ (m^2^/s)	*S_calc_* 10^−5^ (mol/m^3^·Pa)	*S_lit_* 10^−5^ (mol/m^3^·Pa)	Reference
CH_4_	5.0	45.02	1.11	3.15	[45]
CO_2_	35.2	47.07	7.47	43.80	[45]
H_2_S	160.0	46.63	91.62	107.8	[46]
CH_4_	2.5	11.10	2.26	2.04	this work

^a^ 1 Barrer = 3348 × 10^−16^ mol·m/(m^2^·s·Pa).

**Table 4:** Comparison of the gas permeability and selectivity of the supported ionic liquid membranes (SILMs).

IL	Support	Permeability, Barrer			Selectivity		Reference
		CH_4_	CO_2_	H_2_S	CO_2_/CH_4_	H_2_S/CH_4_	
bmim[OAc]	MFFK-1	6.0 ± 0.3	92.0 ± 3.0	115.0 ± 3.0	15.0 ± 0.4	19.0 ± 0.6	this work (pure gases)
bmim[OAc]	MFFK-1	2.0 ± 0.1	186.1 ± 5.7	205.8 ± 6.2	96.9 ± 2.9	102.9 ± 3.1	this work (binary mixture)
bmim[OAc]	MFFK-1	1.0 ± 0.1	100.3 ± 3.0	110.4 ± 3.3	96.5 ± 2.9	106.2 ± 3.2	this work (ternary mixture)
bmim[BF_4_]	MFFK-1	12.0 ± 0.3	84.0 ± 2.0	383.0 ± 12.0	7.0 ± 0.2	32.0 ± 1.0	this work (pure gases)
bmim[BF_4_]	MFFK-1	7.9 ± 0.3	68.9 ± 2.1	128.6 ± 3.9	8.7 ± 0.3	23.4 ± 0.7	this work (binary mixture)
bmim[BF_4_]	MFFK-1	8.6 ± 0.3	70.0 ± 2	142.0 ± 3.0	8.0 ± 0.2	16.4 ± 0.5	this work (ternary mixture)
bmim[OAc]	PVDF	37.2	443	5279	11.9	142	[47] (pure gases)
bmim[BF_4_]	PVDF	92.4	1056	3708	3.5	40	[47] (pure gases)
bmim[BF_4_]	PVDF	4	180	1100	6	260	[50] (pure gases)

## Abbreviations

SILM	supported ionic liquid membrane
bmim[OAc]	1-butyl-3-methylimidazolium acetate
bmim[BF_4_]	1-butyl-3-methylimidazolium tetrafluoroborate
IL	ionic liquid
RTIL	room temperature ionic liquid
emim[BF_4_]	1-ethyl-3-methylimidazolium tetrafluoroborate
emim[dca]	1-ethyl-3-methylimidazolium dicyanamide
emim[CF_3_SO_3_]	1-ethyl-3-methylimidazolium trifluoromethanesulfonate
C_4_mim[NTf_2_]	1-butyl-3-methylimidazolium bistriflamide
DIP-C_4_mim[NTf_2_]	diisopropyl 1-alkyl-3-methylimidazolium bistriflamide
FTM	facilitated transport membrane
PTFE	polytetrafluoroethylene
emim[OAc]	1-ethyl-3-methylimidazolium acetate
MFFK-1	a flat sheet porous tetrafluoroethylene-vinylidene fluoride membrane with pore size equal to 150 nm
SEM	scanning electron microscopy
GC	gas chromatography
GCMS	gas chromatography mass-specrtometry
TCD	thermal conductivity detector
ppm	part per million, 10^−4^%
